# Impact of COVID-19 on hospital visits for non-traumatic dental conditions in Ontario, Canada

**DOI:** 10.1186/s13104-023-06380-5

**Published:** 2023-08-02

**Authors:** Sonica Singhal, Badal Dhar, Nardin Ayoub, Carlos Quiñonez

**Affiliations:** 1 Health Promotion, Chronic Disease and Injury Prevention Department, Public Health Ontario, 480 University Avenue, Suite 300, Toronto, ON M5G 1V2 Canada; 2 Discipline of Dental Public Health, Faculty of Dentistry, University of Toronto, Toronto, ON Canada; 3 Data and Information Management Services, Public Health Ontario, 480 University Avenue, Suite 300, Toronto, ON M5G 1V2 Canada; 4 Department of Health Sciences, Wilfrid Laurier University, Waterloo, ON Canada; 5 Schulich School of Medicine and Dentistry, Western University, London, ON Canada

**Keywords:** Access to care, Emergency department visits, Day surgeries, Hospitalizations, Non-traumatic dental conditions, COVID-19

## Abstract

**Background and rationale:**

As general health care is publicly funded in Canada and oral health care is not, many people seek care from hospitals for their dental problems. This study assessed if the unprecedented times of Coronavirus disease (COVID-19) affected the hospital visits for dental emergencies, making disadvantaged populations further vulnerable for attendance of their dental problems.

**Methods:**

Data from IntelliHealth Ontario for emergency department (ED) visits, day surgery visits, and hospitalizations associated with non-traumatic dental conditions (NTDCs) were retrieved for years 2016 to 2020 to assess trends before COVID-19 and changes, if any, for the year 2020. Trends by month, for the years 2019 and 2020, to make straight comparisons and understand the effects of lockdown in Ontario, was also analyzed.

**Results:**

In the year 2020, there was a reduction of 40% in day surgeries, 21% in ED visits and 8% in hospitalizations compared to 2019. Stratified by month, largest reductions were observed in April 2020: 96% in day surgeries; 50% in ED visits; and 38% reductions in hospitalizations when compared to the same month of 2019. In May 2020, day surgeries and ED visits though remained reduced, hospitalization rates increased by 31%.

**Conclusion:**

Hospital EDs are inefficient avenues for handling dental emergencies. Nevertheless, they do remain a care setting that is sought by many for dental problems, and if the need for hospitalization and day surgery is there, this care setting is an important avenue for dentally related medical care. Perhaps unsurprisingly, COVID-19 has lessened the opportunity and capacity for such care.

**Practical implications:**

Administrators and policy makers can utilize this information to strategize on augmenting community infrastructure for building more effective, and cost-efficient avenues of care for timely management of dental problems.

## Background/introduction

Maintaining good oral health requires timely access to routine oral health care [1]. However, most oral health care in Canada is privately funded through employer or individually sponsored dental insurance, which can make affordability a major barrier to accessing care among uninsured and underinsured populations [2, 3]. As a result, since physician and hospital care are publicly funded in Canada, individuals who experience non-traumatic dental conditions (NTDCs) and cannot afford oral health care services will visit general physicians or hospital emergency departments (EDs) seeking a resolution to their oral health problems [4–6]. Such visits are neither effective nor cost-efficient in Canada since dentists are not available in EDs and physicians do not receive substantial training related to oral diseases and conditions. That said, medical personnel help in emergency situations where NTDCs significantly contribute to morbidity or are life threatening such that hospitalization is required (e.g., an oral infection causing airway obstruction [7]). At worse, medical personnel unnecessarily prescribe analgesics and antibiotics to alleviate the symptoms of NTDCs and provide verbal referrals to those with no recourse to definitive oral health care [8, 9].

Hospital operating rooms, where day surgery is performed under general anaesthesia, is another venue for Canadians to receive care for severe or multiple dental issues that cannot be attended to in routine dental practices [10, 11]. It is important to note that barriers to timely access to routine dental care attribute to dental conditions becoming severe [12]. Studies have shown that dental surgery for early childhood caries is common, especially among children from low-income families, in rural and remote regions, and Indigenous communities [10, 11]. Overall, studies confirm that for NTDCs, hospital EDs or operating rooms are utilized more often by socioeconomically disadvantaged populations [13].

On February 11, 2020, Coronavirus disease (COVID-19) was declared a pandemic by the World Health Organization (WHO) [14]. As the pandemic progressed, not only did it directly affect the health of people, it also impacted the functioning of health care systems [15]. For example, avoiding hospital EDs was a trend observed globally. Zipursky et al. noted a decline of 32% in overall number of ED visits in Ontario’s hospitals from March 2020 to June 2020, comparing to the same time period of 2019 [16]. According to the Canadian Institute for Health Information (CIHI), the biggest drop in ED visits in Canada occurred during April 2020, by almost half the usual number, which was attributed to how people interpreted public health restrictions and their fear of contracting COVID-19 when visiting a hospital [17]. A study in Alberta, Canada, showed 35% reduction in daily ED visits from March 16th to September 23rd 2020 comparing to the same time period of 2019, the probable reason concluded was the contagious nature of the COVID-19 virus [15]. Boserup et al. utilizing data from the National Center for Immunization and Respiratory Diseases (NCIRD) division of the Centers for Disease Control and Prevention (CDC) analyzed that the mean number of ED visits per week for the 4 weeks (March 15th to April 5th 2020) in the United States was significantly less than a period of 4 weeks prior to COVID-19 pandemic. The percentage decrease varied by region ranging from 31 to 45%. The researchers concluded that COVID-19 related fears have resulted in fewer visits to the ED for emergent health conditions [18]. A study from Italy also showed a 25.3% decrease in ED visits in 2020 compared to 2019, with the highest decrease in March 2020, by 52.4% [19]. Similarly, a study from Taiwan witnessed a 33.4% decrease in non-traumatic ED visits during February to April 2020 compared to the same time frame of 2019 [20]. In short, people have ignored or neglected the signs and symptoms of medical conditions for which they would have normally visited an ED [18].

Avoiding health care for conditions that require urgent care result in serious consequences [21]. Deferring care can worsen morbidity and mortality rates, especially for disadvantaged communities that are dependent on EDs for their health problems [22]. A study from California, US, found a significant increase in cardiac arrests where people were declared dead on the scene during the pandemic, suggesting that people were not seeking medical care [21]. Importantly, despite research on the impact of COVID-19 on ED visits for various health conditions, little is understood about ED usage for NTDCs during the pandemic.

This study aims to quantify hospital ED and operating room loads for NTDCs before and during COVID-19 in Ontario, Canada’s most populated province with approximately 39% of the country’s population [23] and to compare it with previous years. As well, given that dental offices in Ontario had to significantly limit their activity in the early stages of the pandemic due to government and regulatory restrictions, many had to wait to access oral health care [24], which could have made their oral conditions worse. As such, this study also aims to compare ED-related hospitalization for NTDCs before and during COVID-19.

## Methods

We analyzed ED visits, day surgery visits, and hospitalizations associated with NTDCs in Ontario for the year 2020. This included discharge diagnoses per International Classification of Diseases (ICD) codes for conditions related to hard tissues of the mouth, specifically teeth and their supporting structures (periodontal tissues and bone). These included dental caries (K02.9), periapical abscess without sinus (K04.9), and tooth ache (K08.87). No soft tissue conditions were included as they could possibly be related to COVID-19 infection.

Data from IntelliHealth Ontario for the years 2016 to 2020 were retrieved by Analytic Services of the institution. Data for 2016–2019, the four previous years to 2020 were assessed to review trends before COVID-19. Kendall’s tau test was conducted to assess any significant changes in trends over the years included. We also assessed these trends by month for the years 2019 and 2020, to make straight comparisons and understand the effects of lockdown in Ontario, which was declared on March 17th 2020 and ended on July 24th 2020, the time period when the province ordered closure of all non-essential services including routine dental care [24].

IntelliHealth Ontario is a knowledge repository of Ontario’s health care system that contains clinical and administrative data collected from various sectors, including hospital services, medical services, and population data. The project was approved by the Research Ethics Board of Public Health Ontario. The protocol number for the approved project is 2021-023.01. No funding was received to conduct this study.

The impact of COVID-19 on utilization of hospital services for NTDCs might vary by age. Therefore, we also explore trends for four age groups, 0–6, 7–18, 19–64 and 65 + years. This stratification was a deliberate attempt to compare data with a previous study assessing trends in hospital services use for NTDCs [4].

## Results

In Ontario, in the year 2020, NTDCs resulted in 42,827 ED visits, 7777 day surgery visits, and 719 hospitalizations. The distribution of these estimates across different age groups and their comparison to previous years is described in the following three sections.

### ED visits

**Fig. 1:**
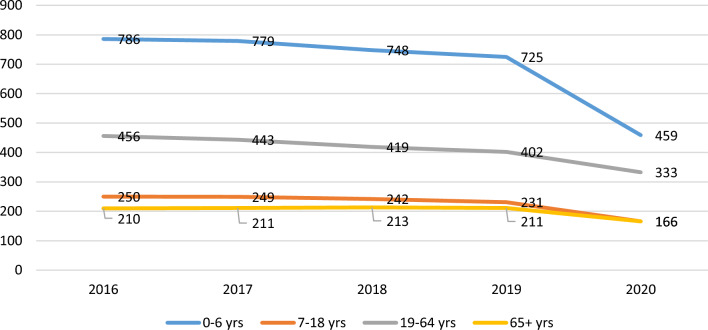
Rate of ED visits per 100,000 in Ontario for dental conditions, by age, for 2016–2020

The rate of ED visits per 100,000 in Ontario for NTDCs, by age, for 2016–2020 is presented in Fig. 1. From 2016 to 2019, the annual average rate of ED visits for NTDCs was 392 per 100,000. The maximum rate, approximately 760 per 100,000 was observed among 0–6 year olds, followed by 430 per 100,000 among 19–64 year olds, 243 per 100,000 among 7–18 year olds, and 211 per 100,000 for those 65 years and above. In terms of trends, a slight insignificant reducing trend was observed in ED visit rates from 2016 to 2019.

In 2020, 291 visits per 100,000 were made to EDs for NTDCs. The rate of visits reduced by 21% in reference to 2019. The reductions were observed among all age groups, with the maximum reduction of 37% observed among 0–6 year olds, followed by 28% for 7–18 year olds, 22% for those 65 years and above, and 17% for 19–64 year olds.

**Fig. 2:**
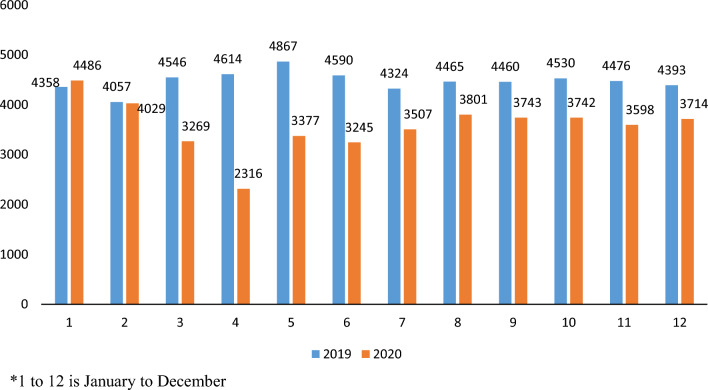
Emergency Department visits in Ontario for Non-Traumatic Dental Conditions, by month, for 2019 and 2020

Observing by month (Table 1), the visits reduced maximum, by 50%, in the month of April 2020, the first full month experiencing the impact of COVID-19 and lockdown. The full trends by each month can be reviewed in Fig. 2.

**Table 1:** Proportion change in ED visits, day surgeries and hospitalizations from 2019 to 2020, stratified by month

Proportion change from 2019 to 2020 (%)	Jan	Feb	Mar	Apr	May	Jun	Jul	Aug	Sept	Oct	Nov	Dec	Total
ED visits	− 3	1	28	50	31	29	19	15	16	17	20	15	20
Hospitalization	8	− 27	16	38	− 31	16	27	− 4	25	− 23	9	8	7
Day surgeries	− 6	− 1	43	96	89	75	42	32	24	24	27	9	39

### Hospitalizations

**Fig. 3:**
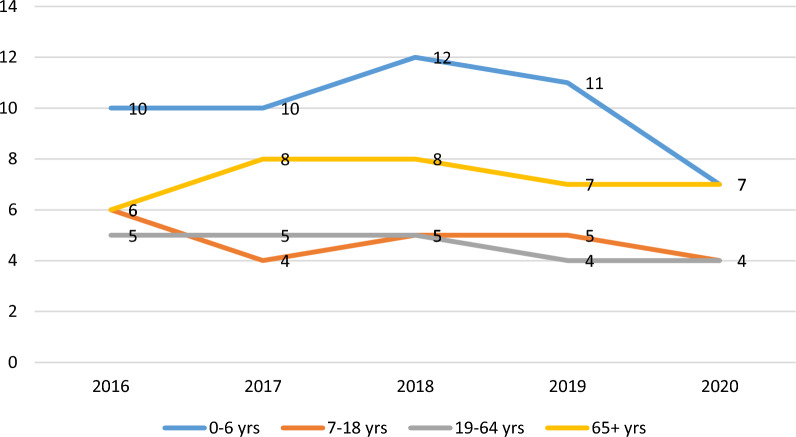
Rate of hospitalizations per 100,000 in Ontario for dental conditions, by age, for 2016–2020

The rate of hospitalizations per 100,000 in Ontario for NTDCs, by age, for 2016–2020 is presented in Fig. 3. From 2016 to 2019, the average annual rate of hospitalization for NTDCs was 6 per 100,000. The maximum rate of 11 per 100,000 was observed among 0–6 year olds, followed by 7 per 100,000 for those 65 years and above, and 5 per 100,000 among 7–64 year olds. There were no significant trends observed in terms of hospitalization rates for the 2016–2019.

In 2020, overall, 5 hospitalizations per 100,000 were observed for NTDCs. The rate of hospitalizations reduced by 8% in reference to 2019, with a maximum reduction of 35% observed among 0–6 year olds, followed by 22% among 7–18 year olds, and 20% among 19–64 year olds. There was an increase in hospitalizations by 2% among those 65 years and above.

**Fig. 4:**
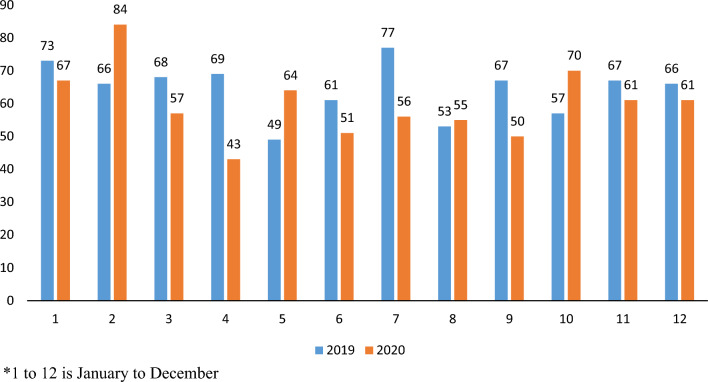
Hospitalizations in Ontario hospitals for Non-Traumatic Dental Conditions, by month, for 2019 and 2020

By month (Table 1), hospitalizations reduced maximum, by 38%, in the month of April 2020; however, the following month, in May 2020, increased by 31%. The full trends by each month can be reviewed in Fig. 4.

### Day surgeries

**Fig. 5:**
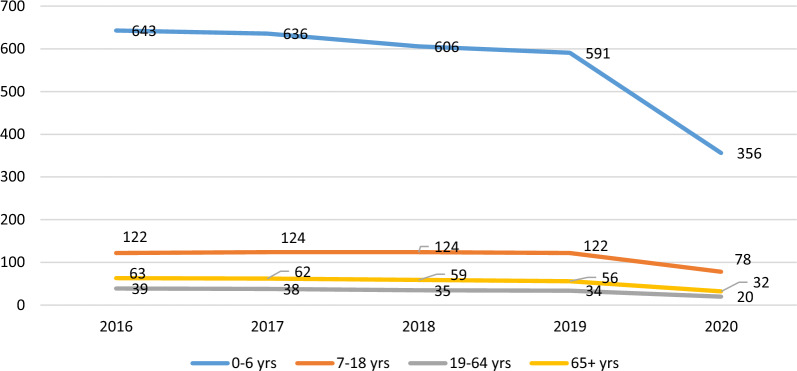
Rate of day surgeries per 100,000 in Ontario for dental conditions, by age, for 2016–2020

The rate of day surgeries per 100,000 in Ontario for NTDCs, by age, for 2016–2020 is presented in Fig. 5. From 2016 to 2019, the annual average rate of day surgeries for NTDCs was 94 per 100,000. The maximum rate of 619 per 100,000 was observed among 0–6 year olds, followed by 123 per 100,000 among 7–18 year olds, 60 per 100,000 among those 65 years and above, and 37 per 100,000 for 19–64 year olds. Again, no significant trends were observed in day surgeries during this time period.

In 2020, 53 day surgeries per 100,000 were observed for NTDCs. The rate of visits reduced by 40% in reference to 2019. Similar reductions were observed among all age groups, with a maximum of 43% among those 65 years and above, followed by 41% for 19–64 year olds, 40% for 0–6 year olds, and 36% for 7–18 year olds.

**Fig. 6:**
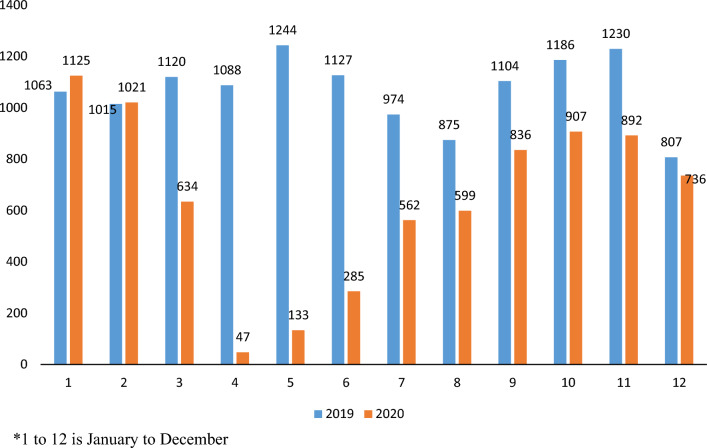
Day surgeries in Ontario hospitals for Non-Traumatic Dental Conditions, by month, for 2019 and 2020

By month (Table 1), day surgeries reduced maximum, by 96%, in the month of April 2020. The full trends by each month can be reviewed in Fig. 6.

## Discussion

In 2020, when COVID-19 was declared a pandemic, the situation created panic and confusion. As hospitals filled with COVID-19 cases, people tried to avoid this health care setting where possible. This has been verified by studies across the globe, which have shown reductions in ED visits for various health conditions because of COVID-19 [16–22, 25]. Our results concur with this general finding and show that the same can be said for NTDCs in Ontario, Canada.

Compared to 2019, in 2020, there was a reduction of 21% and 8% in ED visits and hospitalizations related to NTDCs, respectively. Stratified by monthly data, the month of April experienced the biggest impact, as ED visits reduced by 50% and hospitalizations by 38%; however, it is interesting to observe that in May 2020, the ED visits though remained reduced by 31%, hospitalization rates rather increased 31% compared to the same month of 2019. One can assume that people waited long to get themselves treated for their dental emergencies ending in being hospitalized.

By age group, while ED visits reduced for all, the reduction was greatest among children. This may be attributed to the Healthy Smiles Ontario program, a publicly funded program where any child less than 18 years can receive dental emergency care at a dental office, irrespective of enrollment in the program. One can hypothesize that during the pandemic, parents who were either not aware of the program earlier or did not qualify may have tried accessing care from dental offices through the program instead of through hospitals’ EDs. Future research analyzing administrative data from the program or from similar programs in other jurisdictions can help answer this question.

What is most interesting is that hospitalizations for NTDCs reduced for all age groups except for seniors, or those 65 years and above, where the hospitalization rate increased. Being most vulnerable to COVID-19, maybe seniors were most reluctant to attend EDs for their dental conditions, resulting in greater disease severity and increasing the likelihood of hospitalization.

Hospital day surgeries are recommended for those who cannot be treated routinely in a dental office because of disease severity or behaviour management; these conditions are generally urgent and sometimes emergent too. We found that in Ontario approximately 40% less day surgeries were performed for NTDCs in 2020 compared to 2019 with almost negligible surgeries during the month of April and May. This reduction reflects the lessened capacity for day surgeries within the Ontario hospital system given government and regulatory intervention to safeguard hospital capacity and resources [26]. It also reflects a major barrier to accessing timely oral health care for those vulnerable populations who need care the most.

We do know that hospitals’ EDs are inefficient avenues for handling dental emergencies. Nevertheless, they do remain a care setting that is sought by many for their dental problems, and if the need for hospitalization and day surgery is there, this care setting is an important avenue for dentally related medical care. Perhaps unsurprisingly, COVID-19 has lessened the opportunity and capacity for such care. This is Ontario data but the assumption is that we may have similar observations across Canada.

In the current times, when national dental care plan is being discussed [27], it is important to assess if some proportion of this funding can be used for developing infrastructure to deal with dental emergencies for all. The dental academic institutes can utilize such data to come together and make a case for intramural emergency dental clinics and operating rooms, and also satellite clinics across community settings so that more effective and cost-efficient avenues of continuous dental care are available for all Canadians irrespective of their social strata. It can also provide opportunities to dental students to gain more experience with dental emergencies.

In terms of strengths and limitations of this research work, the data utilized were population-based administrative data, which is not a proxy but shows the actual usage of hospitals during COVID-19 for NTDCs. However, as data collecting, quality checks, and reporting takes time, authors could not access data for the year 2021, which if not more was similarly affected as 2020 by the pandemic, at the time of analysis for this study. This could have provided further insights on trends of NTDCs associated visits to hospitals.

## Limitations of this work

These results show the utilization of emergency and acute care services based on administrative data; there is no information who faced the barriers in accessing care the most and how patients coped with their oral conditions.

This study did not include data for years 2021 and 2022, as the intent was to examine the immediate effect of pandemic; however it would be interesting to assess the future trends of healthcare system utilization for NTDCs, not only to understand how things changed post-pandemic, but also to assess the impact of Canada Dental Benefit and the proposed national dental care program.

## Conclusion

With the declaration of COVID-19 as a pandemic in March 2020, a remarkable reduction in hospitals visits for NTDCs was observed in Ontario. There is no information how these patients coped with their dental conditions; however, there is enough data to show that hospitals in general are sought by many for their dental and dental related health conditions. Unavailability of hospital services during COVID-19 highlighted vulnerabilities of the disadvantaged populations. This should be an awakening for health care system administrators and policy makers to strategize on how to build community infrastructure so that more effective and cost-efficient avenues of care are available for NTDCs in times of crisis and in more peaceful times as well. Getting treated for oral pain and infection without barriers to care is the bare minimum every Canadian should expect.

## Data Availability

Administrative data are available through Intellihealth Ontario.

## Abbreviations

CIHI: Canadian Institute for Health Information
CDC: Centers for Disease Control and Prevention
COVID-19: Coronavirus disease
ED: Emergency department
ICD: International Classification of Diseases codes
NCIRD: National Center for Immunization and Respiratory Diseases
NTDCs: Non-traumatic dental conditions

## References

[1] Canadian Dental Association (CDA). Oral Health — Good for LifeTM: http://www.cda-adc.ca/en/oral_health/cfyt/good_for_life/. Accessed 10 Feb 2022

[2] Thompson B, Cooney P, Lawrence H, Ravaghi V, Quiñonez C (2014). The potential oral health impact of cost barriers to dental care: findings from a Canadian population-based study. BMC Oral Health.

[3] Cooney P, Deonandan R, Burstyn I, Sithole F, Zwaigenbaum L, Colquhoun A, Jiang Z, Maiangowi G, Ashbury F, Chen YDW (2009). Preface to the Canadian health measures survey results—oral health statistics 2007–2009. Oral Health.

[4] Singhal S, Quiñonez C, Manson H (2019). Visits for nontraumatic dental conditions in Ontario’s health care system. JDR Clin Transl Res.

[5] LaPlante NC, Singhal S, Maund J, Quiñonez C (2015). Visits to physicians for oral health-related complaints in Ontario, Canada. Can J Public Health.

[6] Figueiredo R, Fournier K, Levin L (2017). Emergency department visits for dental problems not associated with trauma in Alberta Canada. Int Dent J.

[7] Silva CM, Paixão J, Tavares PN, Baptista JP (2021). Life-threatening complications of ludwig’s angina: a series of cases in a developed country. BMJ Case Rep.

[8] Okunseri C, Okunseri E, Xiang Q, Thorpe JM, Szabo A (2014). Prescription of opioid and nonopioid analgesics for dental care in emergency departments: findings from the national hospital ambulatory medical care survey. J Public Health Dent.

[9] Rm R, Al H, Dj S, F-D KE, LA H (2019). Antibiotic prescriptions associated with dental-related emergency department visits. Ann Emerg Med.

[10] Schroth RJ, Quiñonez C, Shwart L WB. TREATING EARLY CHILDHOOD CARIES UNDER GENERAL ANESTHESIA: A NATIONAL REVIEW OF CANADIAN DATA - PubMed. J Can Dent Assoc: https://pubmed.ncbi.nlm.nih.gov/27548666/. Accessed 10 Feb 2022 27548666

[11] Canadian Institute for Health Information. Treatment of preventable dental cavities in preschoolers: a focus on day surgery under general anesthesia. CIHI. 2013:1–34.

[12] Canadian Academy of Health Sciences| Académie canadienne des sciences de la santé. Improving access to oral health care for vulnerable people living in Canada: https://cahs-acss.ca/improving-access-to-oral-health-care-for-vulnerable-people-living-in-canada/. Accessed 20 Feb 2022

[13] VanMalsen JR, Figueiredo R, Rabie H, Compton SM (2019). Factors associated with emergency department use for non-TRAUMATIC dental problems: scoping review. J Can Dent Assoc.

[14] World Health organization (WHO). WHO Director-General’s remarks at the media briefing on 2019-nCoV on 11 February 2020: https://www.who.int/director-general/speeches/detail/who-director-general-s-remarks-at-the-media-briefing-on-2019-ncov-on-11-february-2020. Accessed 10 Feb 2022

[15] Rennert-May E, Leal J, Thanh NX, Lang E, Dowling S, Manns B (2021). The impact of COVID-19 on hospital admissions and emergency department visits: a population-based study. PLoS ONE.

[16] Zipursky JS, Stall NM, Silverstein WK, Huang Q, Chau J, Hillmer MP (2021). Alcohol sales and alcohol-related emergencies during the COVID-19 pandemic. Ann Intern Med.

[17] Canadian Institute for Health Information (CIHI). COVID-19’s impact on emergency departments: https://www.cihi.ca/en/covid-19-resources/impact-of-covid-19-on-canadas-health-care-systems/emergency-departments. Accessed 17 Feb 2022

[18] Boserup B, McKenney M, Elkbuli A (2020). The impact of the COVID-19 pandemic on emergency department visits and patient safety in the United States. Am J Emerg Med.

[19] Bardin A, Buja A, Barbiellini Amidei C, Paganini M, Favaro A, Saia M (2021). Elderly people’s access to emergency departments during the COVID-19 pandemic: results from a large population-based study in Italy. J Clin Med.

[20] Tsai L-H, Chien C-Y, Chen C-B, Chaou C-H, Ng C-J, Lo M-Y (2021). Impact of the coronavirus disease 2019 pandemic on an emergency department service: experience at the largest tertiary center in Taiwan. Risk Manag Healthc Policy.

[21] WongLaura E (2020). Where are all the patients? addressing Covid-19 fear to encourage sick patients to seek emergency care. Catalyst nejm org.

[22] Nourazari S, Davis SR, Granovsky R, Austin R, Straff DJ, Joseph JW (2021). Decreased hospital admissions through emergency departments during the COVID-19 pandemic. Am J Emerg Med.

[23] Statistics Canada. The Daily—Canada’s population estimates, second quarter 2021: https://www150.statcan.gc.ca/n1/daily-quotidien/210929/dq210929c-eng.htm?indid=4098-1&indgeo=0. Accessed 10 Feb 2022

[24] Government of Ontario. Report on Ontario’s Provincial Emergency from March 17, 2020 to July 24, 2020 | Ontario.ca: https://www.ontario.ca/document/report-ontarios-provincial-emergency-march-17-2020-july-24-2020. Accessed 20 Feb 2022

[25] Gerhold L. COVID-19: Risk perception and Coping strategies. 2022: https://psyarxiv.com/xmpk4/. Accessed 10 Feb 2022

[26] Canadian Institute for Health Information (CIHI). COVID-19’s impact on hospital services: https://www.cihi.ca/en/covid-19-resources/impact-of-covid-19-on-canadas-health-care-systems/hospital-services. Accessed 21 Feb 2022

[27] Singhal S., Quinonez C. 7 principles to guide a national dental care program in Canada Published: May 2, 2022. https://theconversation.com/7-principles-to-guide-a-national-dental-care-program-in-canada-182267. Accessed 20 July 2022

